# Feasibility of detecting aortic stenosis with mobile phone auscultation data: a pilot study

**DOI:** 10.3389/fcvm.2026.1768473

**Published:** 2026-03-04

**Authors:** Ryan M. Close, Gregory L. Judson, Jacob Zhang, Kailey Kowalski, Destiny Martinez, Marco Diaz, Martin Huecker

**Affiliations:** 1 Department of Medicine, Division of General Internal Medicine, MaineHealth, Portland, ME, United States; 2 Tufts University School of Medicine, Tufts University, Boston, MA, United States; 3 Department of Medicine, Division of Cardiology, MaineHealth, Portland, ME, United States; 4 Department of Internal Medicine Residency Program, MaineHealth, Portland, ME, United States; 5 Department of Echocardiography, Division of Cardiology, MaineHealth, Portland, ME, United States; 6 Department of Emergency Medicine, University of Louisville School of Medicine, Louisville, KY, United States

**Keywords:** aortic stenosis, mitral regurgitation, mobile phones, noninvasive screening, valvular heart disease

## Abstract

**Background:**

The prevalence of valvular heart disease is increasing. Early detection remains poor as screening relies on front line detection of audible or symptomatic disease and confirmation requires specialized echocardiography.

**Methods:**

We conducted a single center, observational pilot study. Eligible subjects were stratified into groups based on echocardiographic findings. In addition to chart extraction of demographics, medical history, and echocardiographic parameters, each subject underwent three auscultation recordings that were analyzed via computational nonlinear dynamics to extract features and construct predictors without fitting or weighting. Predictors were used to create logistic regression binary classification models. Training and test set performance was reported for each model with a focus on area-under-the-curve and sensitivity as the primary benchmarks.

**Results:**

We analyzed the recordings of 248 subjects, median age 73 years, 43.6% female, 99% White. All recordings were chaotic and of low dimensionality. Personnel and subject collected recordings had a normalized mutual information entropy of 1.0, indicating they shared the same information and could be interchangeable for model development. Three models for aortic stenosis met predetermined metrics, with the best performing model reporting an AUC of 0.872 and a sensitivity of 0.923. Mitral regurgitation models were explored but limited by sample size.

**Conclusions:**

This study established the feasibility of two innovative approaches, by combining the sound recordings collected from unmodified mobile phones with analysis via nonlinear dynamics software. This work has the potential to improve valvular heart disease detection by overcoming barriers that remain for current standards of care and emerging artificial intelligence solutions.

## Introduction

Valvular heart disease (VHD) remains an underappreciated and growing public health concern, currently affecting over 13% of the population 75 years and older and is a major contributor to preventable mortality in the aging population of the United States (1).

Aortic stenosis (AS) and mitral regurgitation (MR) represent two of the most common valvular lesions in the U.S. Both conditions are significantly underdiagnosed, increase in prevalence with age, and have prolonged asymptomatic periods with variable natural histories. The primary means of reducing morbidity and mortality depend on consistent monitoring and early intervention, when applicable (2, 3).

The diagnostic pathway of valvular disease involves a combination of symptoms and physical findings, which are both imperfect and nonspecific (4, 5). Auscultation with stethoscopes remains the primary tool to screen for VHD and therefore largely limits clinicians to disease that is audible. In addition to inter-provider variability in the auscultation and classification of audible cardiac disease (e.g., murmurs), confirmation and monitoring requires echocardiography, the accepted gold standard (6) which necessitates specialized equipment, and highly trained personnel. Additional barriers to diagnostic care include access, transportation, health literacy, and insurance coverage, which perpetuate a system of late and undiagnosed VHD.

Screening, diagnosis, and monitoring of VHD would greatly benefit from novel technologies that do not overly rely on the availability of advanced imaging or subspecialists. Basic mobile phones contain sensitive recording devices, improving computational power, and if paired with accurate modeling software, could potentially address enumerable existing barriers to screen or monitor patients with VHD. We conducted a pilot study using auscultation recordings collected via unmodified mobile phones and analyzed using nonlinear dynamics software to develop accurate models fitted to echocardiographic findings for aortic stenosis and mitral regurgitation.

## Methods

### Setting and population

We conducted a single center, open label pilot study that utilized convenience sampling at our institution's outpatient echocardiography suite.

### Eligibility criteria

Eligible subjects presented for transthoracic echocardiograms as part of routine clinical care. Subjects who met the following inclusion criteria were approached for enrollment: ≥50 years old, English speaking, and one of the following findings on echocardiogram (group stratification): (1) isolated, unrepaired aortic stenosis of any severity (mild, moderate, or severe); (2) isolated, unrepaired mitral regurgitation (moderate or severe only); (3) structural heart disease other than and excluding aortic stenosis or mitral regurgitation; (4) Controls, defined as those with no findings or those consistent with normal for age. Subjects with aortic stenosis or mitral regurgitation had no disease of other valves nor mixed pathology of valve of concern. For example, subjects with isolated aortic stenosis had no mitral disease nor aortic regurgitation.

Exclusion criteria included persons <50 years of age, pregnancy, incarceration, history of valvular repair or replacement, pacemaker or defibrillator, rheumatic valvular disease, congenital heart disease, or the presence of moderate to severe disease of both aortic and mitral valves.

### Procedures—recordings and echocardiograms

Eligible subjects were pre-screened prior to arrival in the echocardiography suite. Subjects only included patients who were scheduled for complete transthoracic echocardiogram as part of on-going clinical care. All echocardiograms were comprehensive and in accordance with existing guidelines (7, 8). Reading cardiologists were not aware of study involvement as image collection occurred prior to enrollment and reading occurred separately. Echocardiograms and study related mobile phone auscultation occurred the same day.

After sonography was completed, subjects were approached by study staff. Enrollers performed auscultation (i.e., audio recording) utilizing a study specific application (provided by study sponsor TSI) and the original equipment manufacturer (OEM) microphone of a basic unmodified mobile phone. The “dummy” application on the phone was exclusively used for collection and deposition of recordings into study folders. No analyses or modification of the sound was performed by the phone or application. Personnel placed the phone's microphone against the patient's skin to collect two 30-second recordings, one from the aortic site (right second intercostal space) and one from the left mid-axillary region. Next, the subject followed specific instructions to obtain a subject-collected third recording at the aortic site. All audio files were stored in an encrypted, cloud-based server (Box™) accessible only to study personnel.

### Data elements and storage

In addition to auscultation recordings, personnel had access to the electronic medical record for chart extraction of clinical variables: demographics (age, race, sex and ethnicity), medical information (height, weight, body mass index, body surface area), cardiopulmonary co-morbidities, and echocardiogram measurements including ejection fraction, diastology metrics, valvular data and chamber dimensions. Data was stored in a study database on a secure server (REDcap). For details on specific data elements extracted, please see supplementary Tables 1, 2. All echocardiograms were performed by sonographers with specialized training in cardiovascular imaging and reviewed by cardiologists per standard of care. The study protocol included an additional review of all imaging by a second sonographer and cardiologist to ensure accuracy and consistency of data.

**Table 1:** Demographics and medical history of all subjects (*N* = 248), stratified by enrollment group.

Variable	All	Aortic stenosis	Mitral Regurgitation	Structural heart disease, Other	Controls	*p*
Subjects—*N* (%)	248 (100)	69 (27.8)	24 (9.7)	73 (29.4)	82 (33.1)	-
Mild		14 (20.3)	-			-
Moderate		37 (53.6)	21 (87.5)			-
Severe		18 (26.1)	3 (12.5)			-
Age (years)—median (IQR)	73 (64–78)	77 (69–82)^b^	77 (69–81)^b^	73 (63–78)	67 (63–76)	<0.01
Female Sex—*n* (%)	108 (43.6)	27 (39.1)	10 (41.7)	33 (45.2)	38 (46.3)	0.82
Not Hispanic or Latino—*n* (%)	246 (99.2)	68 (98.6)	24 (100)	72 (98.6)	82 (100)	0.68
Race						
White	246 (99.2)	69 (100)	24 (100)	71 (97.3)	82 (100)	0.18
Non-White	2 (0.8)	0 (0)	0 (0)	2 (2.7)^a^	0 (0)	-
Height (cm)—median (IQR)	170.2 (162.6–177.9)	172.7 (162.6–177.8)	169.1 (162.5–172.7)	172.7 (162.6–178.0)	170.2 (165.1–180.3)	0.64
Weight (kg)—median (IQR)	83.9 (70.2–98.8)	85.7 (74.8–99.8)	76.7 (66.6–85.1)	86.6 (70.0–103.0)	82.1 (69.9–96.6)	0.08
Body Surface Area (m^2^)—median (IQR)	1.98 (1.78–2.15)	2.00 (1.84–2.15)	1.88 (1.75–2.00)	2.04 (1.77–2.19)	1.95 (1.78–2.14)	0.15
Body Mass Index (kg/m^2^)—median (IQR)	28.6 (25.0–32.5)	29.7 (25.6–33.7)	25.6 (23.7–28.7)	28.7 (25.1–33.2)	27.9 (25.1–32.2)	0.07
BMI Category—*n* (%)						
Normal	62 (25.0)	14 (20.3)	10 (41.7)	18 (24.7)	20 (24.4)	0.33
Overweight	86 (34.7)	22 (31.9)	9 (37.5)	25 (34.3)	30 (36.6)	
Obese	100 (40.3)	33 (47.8)	5 (20.8)	30 (41.1)	32 (39.0)	
*Class I*	*62* (*25.0)*	*22* (*31.9)*	*3* (*12.5)*	*16* (*21.9)*	*21* (*25.6)*	-
*Class II*	*25* (*10.1)*	*8* (*11.6)*	*1* (*4.2)*	*9* (*12.3)*	*7* (*8.5)*	
*Class III*	*13* (*5.2)*	*3* (*4.4)*	*1* (*4.2)*	*5* (*6.9)*	*4* (*4.9)*	
Cardiopulmonary Comorbidities—*n* (%)						
Mean (SD)	3.3 (2.0)	3.1 (1.8)	3.7 (2.8)	4.1 (2.0)	2.7 (1.8)	0.21
Median (IQR)	3 (2–4)	3 (2–4)	3.5 (1–6)	4 (3–5)^b^	3 (1–4)	<0.01
None	14 (5.7)	1 (1.5)	3 (12.5)	2 (2.7)	8 (9.8)	0.04
≥1	234 (94.4)	68 (98.6)	21 (87.5)	71 (97.3)	74 (90.2)	0.04
≥3	163 (65.7)	46 (66.7)	15 (63.5)	60 (82.2)^b^	42 (51.2)	<0.01
≥5	57 (23.0)	11 (5.9)	8 (33.3)^b^	29 (39.7)^b^	9 (11.0)	<0.01
≥7	20 (8.1)	6 (8.7)	5 (20.8)	6 (8.2)	3 (3.7)	0.06

^a^ Two subjects identified as a non-White race, 1 Asian and 1 Black/African American.

^b^ indicates that *p*-value met level of statistical significance (*p* < 0.017) in pairwise comparison with healthy controls.

**Table 2:** Biofluid dynamic model performance characteristics predicting categorical or continuous echocardiogram variables for aortic stenosis and mitral regurgitation.

Model	AS1	AS2	AS3	MR1
Valve, Condition	Aortic stenosis	Aortic stenosis	Aortic Stenosis	Mitral Regurgitation
Dependent variable	Binary, Diagnosis, as determined by a cardiologist	Binary, Diagnosis, as determined by a cardiologist	Continuous, aortic valve area indexed to body surface area	Binary, Diagnosis, as determined by a cardiologist
Total cases—*n*	248	248	244	245
Validation Set—*n*	157	157	155	156
80% sensitivity	1.000	0.956	0.929	0.710
80% specificity	0.821	0.857	0.821	0.894
80% AUC^a^	0.911	0.906	0.875	0.802
20% sensitivity	1.000	1.000	1.000	0.867
20% specificity	0.929	0.893	0.929	1.000
20% AUC	0.964	0.946	0.964	0.933
Training Set—*n*	196	196	193	195
Sensitivity	1.000	0.964	0.943	0.740
Specificity	0.843	0.864	0.843	0.815
AUC	0.921	0.914	0.893	0.828
Test Set—*n*	52	52	51	50
Sensitivity	0.846	0.923	0.857	0.667
Specificity	0.897	0.821	0.784	0.759
AUC	0.872	0.872	0.820	0.713
True Positive	11	12	12	14
False Positive	4	7	8	7
True Negative	35	32	29	22
False Negative	2	1	2	7
Accuracy	0.885	0.846	0.804	0.720

^a^ Area under the curve.

### Sample size

Study objectives did not include hypothesis testing; thus, sample size was driven by the number of subjects needed to establish Phase I model feasibility. We used a heuristic approach, guided by explicit assumptions, and leveraged the dimensionality of the reconstructed phase space to conservatively estimate number of predictors needed given the size of the data. Prior work using similar methodology has consistently reported a dimension correlation (DCorr) ≤ 2.5 (9, 10). Treating 2.5 as an upper bound on DCorr, we conservatively assumed a maximum of five effective degrees of freedom for model development. Following Peduzzi's guidance for logistic regression to reduce overfitting—requiring at least 10 outcome events per degree of freedom) (11)—a minimum of 50 outcome events were required for training-set model development per condition of interest (e.g., AS, MR). Because the cohort was divided 80% Training set and 20% Test set, an additional 15 subjects per condition were needed for the Test set, yielding a total of 65 subjects per condition. With four outcome groups of interest (AS, MR, other SHD, Controls), the target enrollment was 260 subjects. There was no sub-stratification requirement for disease severity among subjects with AS or MR. At this phase, the intent was to establish feasibility of creating models that categorically identify the presence or absence of a disease state, irrespective of severity.

### Statistical methods

Patient demographics and medical history were summarized using descriptive statistics, including totals and proportions for categorical data and means (standard deviations) or medians (interquartile range) where appropriate for continuous variables. Comparisons were limited to diseased groups (AS, MR, Other SHD) vs. healthy controls using parametric and non-parametric tests, where appropriate, to evaluate significant inter-group differences. For all analyses, an omnibus test was first performed to determine the presence of inter-group differences, followed by individual pairwise testing. In the case of categorical variables, a chi-squared test was performed initially followed by individual chi-squared testing. For normally distributed continuous variables, a one-way analysis of variance (ANOVA) was performed followed by pairwise *t*-test. And for non-normally distributed variables, a Kruskal–Wallis test was followed by Mann–Whitney testing. For the applicable omnibus test a *p*-value of <0.05 was considered significant, with a Bonferroni correction of 3 applied to pairwise testing for a *p*-value of 0.017 to determine significance. All statistical analyses were performed using Stata/SE 18.0 (StataCorp LLC, College Station, TX).

### Model development

After study completion and quality review of all clinical data and sound recordings, study personnel provided deidentified recordings and selected variables to Fleming Scientific, sponsor subcontractor, for model development. Mobile phone auscultation (MPA) recordings consisted of 30-second clips of the aortic and axillary auscultation sites (three per subject). All recordings were analyzed by Fleming Scientific using a physics-based approach known as computational nonlinear dynamics (NLD) or Time Series Dynamics (TSD). Similar methodology has been utilized to develop models for COVID diagnosis (12), low ejection fraction (10), volume overload (13), and chronic obstructive pulmonary disease (COPD) (9).

All recordings were initially evaluated for low dimensional chaos as evidenced by maximal Lyapunov exponent (MLE) and correlation dimension (DCorr). Recordings were clustered according to demographics, body measurements (e.g., BMI, BSA), and nonlinear variables (MLE, DCorr) without knowledge of study grouping. Within each cluster, the study principal investigator randomly assigned recordings to two groups: 80% training set and 20% to the test set, ensuring a proportional balance in diagnoses between the two sets. Fleming Scientific was then provided the training set for model development without test set knowledge.

Features were then extracted via two different processes. First, nonlinear dynamic processes were used that included various approaches to fractal dimensions as well as research quantities such as MLE, correlation dimension, Hurst exponent, Rényi and Sample entropy. Second, common acoustic signal analysis processes were used that included power spectrum and cepstrum. Variables from both processes along with subject specific variables (age, sex, body mass index, and body surface area) were all eligible features as part of model development.

Next, Kolmogorov–Smirnov testing was used to confirm that the most relevant features used in test and train sets likely came from the same universe. A validation, or tuning, set was created from 80% of the training set. Using a metaheuristic method, features were elected and combined into three “predictors” without weighting or fitting. Features were extracted from the recordings which are chaotic time series by using domain appropriate nonlinear dynamics outlined above. Several optimization and metaheuristic settings were used with minimum entropy providing the best results. The intent of this combinatorial approach was to place the weight of the model on the extracted features and not on the fitting. This intent allows for preservation of the physics of the model as much as possible and *minimize* fitting necessary to achieve target results. Within the context of metaheuristic optimization, features were selected, scaled and added together to create proforma predictors. The most promising combinations of three predictors were then fitted with logistic regression to the dependent variable (depending on the model and dependent variable of choice) using only the tuning set. The number of predictors (three) is guided by the dimensionality of the phase space and the size of the data se per Peduzzi simulation.

**Figure 1:**
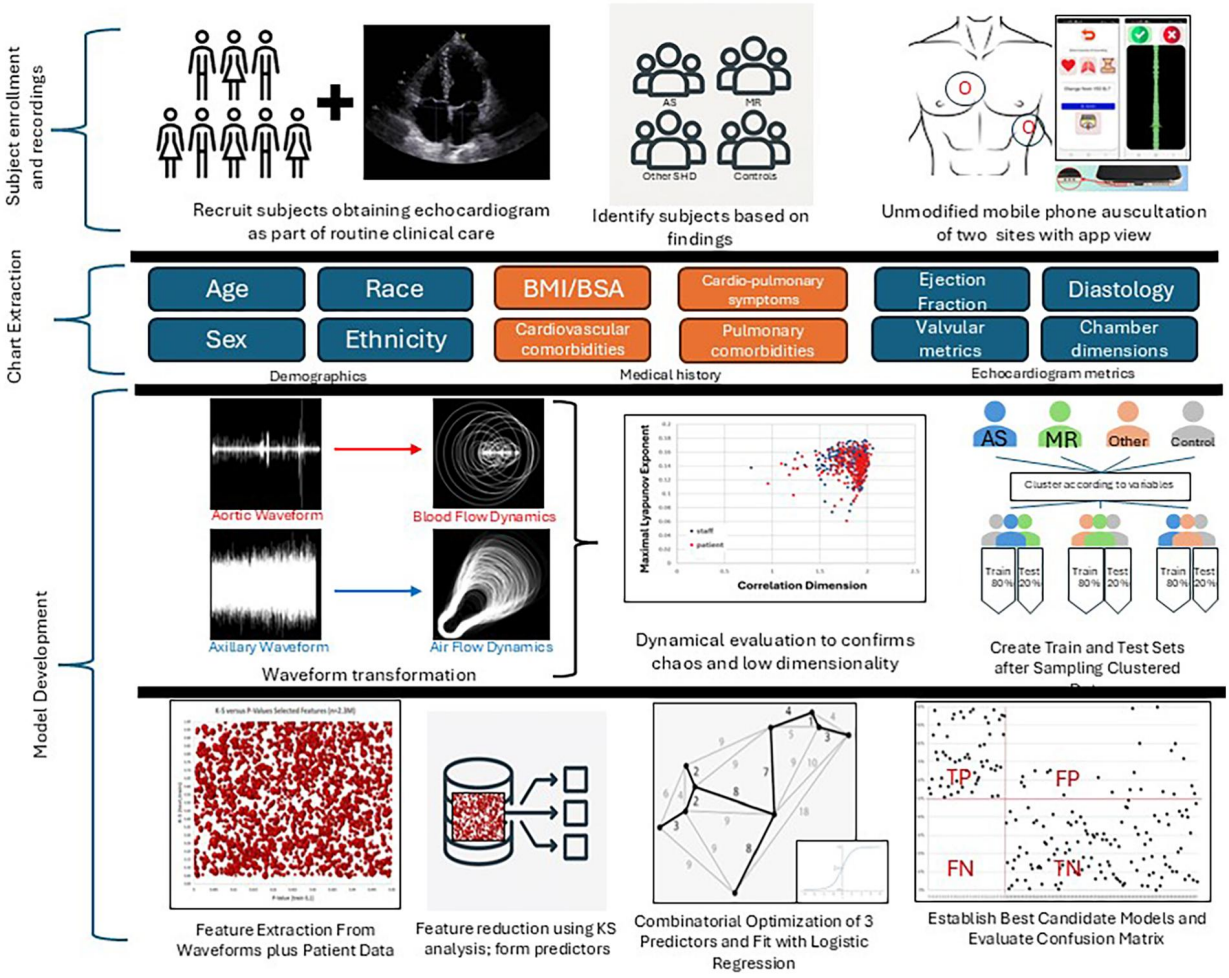
Data collection methodology and model development process.

No knowledge of the test set was used. After identification of tuning models, both the training and test sets were passed through them. Evaluation placed primary consideration for test set area-under-the-curve (goal > 0.800) and sensitivity (goal > 0.85). See Figure 1 for full methodology overview. Binary models were developed to predict parameters of gold standard echocardiographic outcomes (dependent variables) for aortic stenosis and mitral regurgitation. Although many features proved to be statistically significant, all of the features, in all the best performing models, came entirely from nonlinear dynamics. The performance characteristics (sensitivity, specificity, area under the curve, and accuracy) of the validation, training, and test sets were provided for each model.

### Ethics

All study procedures were reviewed and approved by the MaineHealth Institutional Review Board (IRB #2189596).

## Results

### Subjects

From December 1, 2024 to March 20, 2025, 257 eligible subjects were enrolled in the study. Nine (3.5%) were subsequently excluded from analysis due to phone malfunction (unrelated to application or recording collection) for at least one of the three recordings, leaving 248 subjects with analyzable recordings. No recordings or subjects were excluded on sound quality, noise interference, user-error in collection, or for similar reason. Sixty-nine (27.8%) had aortic stenosis, 24 (9.7%) had moderate to severe mitral regurgitation, 73 (29.4%) had other SHD, and 82 (33.1%) met criteria as healthy controls (Table 1). Median age of the cohort was 73 years (IQR 64–78) and 108 (43.6%) were female.

Subjects with AS and MR were older than controls: 77 years vs. 67 years, respectively. Nearly all participants were White (>99%) and a significant proportion were overweight (34.7%) or obese (40.3%), with no significant inter-group differences. Cardiopulmonary co-morbid conditions were common, with a median of 3 (IQR 2–4) conditions per subject. Subjects in the Other-SHD group had more comorbidities compared to controls (Table 1). Only 14 (5.7%) subjects had no cardiopulmonary comorbidities. For details on the distribution between groups, see Supplementary Table S3.

### Model results

All recordings were chaotic (MLE > 0) and of low dimensionality (DCorr < 3), which justified the modeling approach. Recordings of the aortic auscultation site collected by study staff and subjects were similar on this basis (Supplementary Figure S1). Evaluation of the normalized mutual information (NMI) entropy between personnel and subject collected recordings was 1.0, indicating the recordings (personnel and subject collected) likely contained the same information.

### Aortic stenosis

Analysis yielded three acceptable models for aortic stenosis. Two models were mapped to the binary dependent variable “Diagnosis of aortic stenosis as determined by a cardiologist”. Validation set AUC for both models exceeded 0.900. Model AS1 test set had a sensitivity, specificity, and AUC of 84.6%, 89.7%, and 87.2%, respectively. Model AS2 test set had a sensitivity, specificity, and AUC of 92.3%, 82.1%, and 87.2%, respectively. For both models, the AUC drop off from training set to test set was 4.9% and 4.2%, respectively (Table 2).

A third model was developed for aortic stenosis (AS3) mapping features to aortic valve area indexed to body surface area (AVAi), a continuous dependent variable. Model test set sensitivity, specificity, and AUC were 85.7%, 78.4%, and 82.0% respectively. See Table 2.

### Mitral regurgitation

We did not meet enrollment targets for mitral regurgitation, arriving at only 24 of the necessary 65 subjects with moderate to severe MR. Of the 224 remaining subjects, 70 had “mild” mitral regurgitation. The majority of these cases were diagnosed qualitatively through jet visualization. We attempted to develop a fourth model (MR1, Table 2) using a binary categorical dependent variable of “mitral regurgitation as determined by a cardiologist”. Test set results did not meet *a priori* targets, with a sensitivity of 66.7% and AUC 71.3%.

## Discussion

This pilot established the feasibility of two novelties: utilizing basic mobile phones with unmodified original microphones for the collection of sound data, evaluating what may be considered a near-universally available recording device given the ubiquity of mobile devices. Second, the potential utility of a dynamical approach to analyzing that sound data for an overall improved model performance. The study met predetermined goals in creating three acceptable models for aortic stenosis using auscultation recordings collected by unmodified mobile phones and analyzed with nonlinear dynamics software. In aortic stenosis modeling, the collective results using either binary (presence or absence of a diagnosis of aortic stenosis) or continuous dependent variables (indexed aortic valve area), yielded a sensitivity, specificity, and accuracy as high as 92.3%, 89.7%, and 88.5%, respectively. Based strictly on performance metrics, the categorical diagnostic model (AS2, Table 2) performed best in terms of AUC (0.872) and sensitivity (0.923).

Given insufficient enrollment of moderate-to-severe MR, a *post-hoc* exploratory analysis incorporating mild MR was performed to evaluate signal detectability across a broader disease spectrum. The *post-hoc* MR model still did not meet predefined performance targets for sensitivity and AUC, reflecting both limited sample size and increased heterogeneity in disease characterization, and had a sensitivity and specificity of 66.7% and 75.9%, respectively. These results should be interpreted as hypothesis-generating only. Unlike aortic stenosis, mitral regurgitation severity was frequently determined using qualitative echocardiographic assessment based on jet appearance rather than a single continuous quantitative parameter. This likely introduced label variability, particularly among mild cases, which may have attenuated model performance.

**Figure 2:**
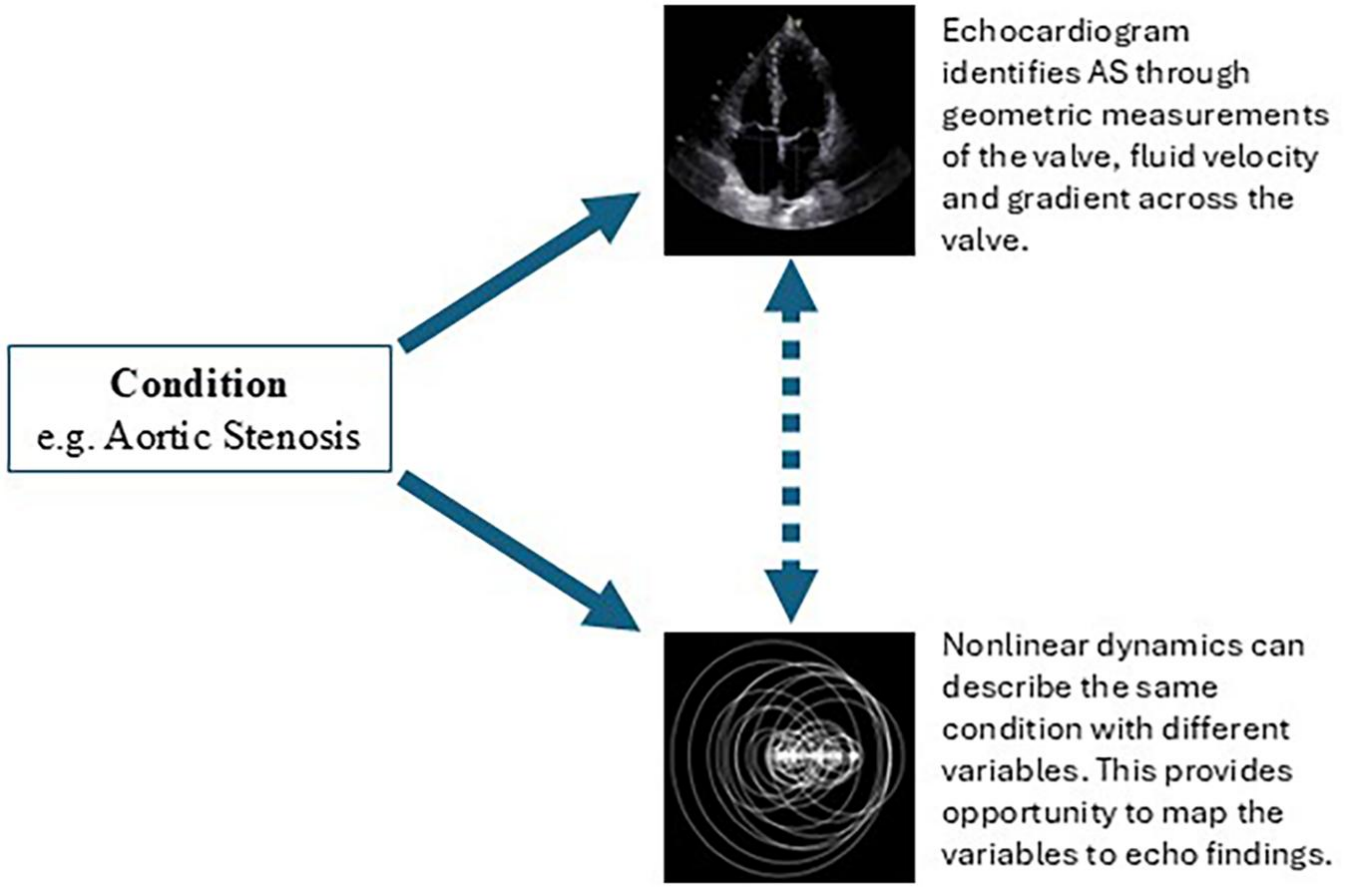
Schematic—nonlinear dynamic models can recreate gold standard findings without necessarily reproducing those findings by describing the same physical state in a unique way.

To be clear, the performance of our modeling only reflects the feasibility of the approach that uses mobile phones and nonlinear features. Further, for the purposes of this study, feasibility was established for isolated aortic stenosis only. Model performance should not be interpreted as clinical applicable or accurate and further research will be necessary to understand model development in mixed valvular disease (aortic stenosis with concomitant aortic insufficient and/or mitral disease). We recognize that classical linear features may also be appropriate for modeling VHD and further appreciate that our work needs to be validated in larger cohorts with differing prevalence estimates. Yet we do not lose sight of the importance of exploring new modeling methods and believe our work contributes a great deal. Further, the models described in this study do not intend to reproduce echocardiogram findings, a biophysically different modality for the detection of conditions. Rather, these nonlinear dynamic models likely reflect turbulence, the fluid dynamics of the specified condition. This method is another means of detecting the same condition using different variables which are then mapped to *predict* echocardiogram findings (see Figure 2).

As outlined in our methods, the models were derived through interrogation of different features (NLD variables, standard acoustics, and subject data). While some non-NLD features (e.g., gender, age) were found to be statistically significant in KS analysis, only NLD variables survived the modeling. Thus, all the features used in the combinatorial optimization process of model development came from **nonlinear dynamics extraction**. Although auscultation-based screening tools are often sensitive to body habitus and acoustic attenuation, the nonlinear dynamics features used here primarily reflect low-frequency hemodynamic turbulence, which propagates efficiently through tissue and is less dependent on surface acoustic transmission. Consistent with this, subject-level demographic and anthropometric variables were not retained in final models. Nonetheless, validation in larger and more diverse populations remains necessary to confirm generalizability. This makes our modeling process unique when compared to what is represented elsewhere in literature. Based on the calculations used, all the models likely represent specific types of turbulence that reflect stress caused by organ dysfunction and compensation. However, this study was not designed to verify the physiological nature of the features, only their modeling utility.

No recordings, collected by either study personnel or study subjects, were rejected secondary to sound quality, interference, or similar reason. Further, the analysis of the separately collected recordings supported the notion that they observations of the same system, and that self-recordings (e.g., remote monitoring) is feasible as well.

A noninvasive means of screening, diagnosing, and monitoring VHD has significant potential implications. The largest contemporary mortality review of patients with untreated aortic stenosis found that only 60% of patients with severe disease underwent treatment within 4 years of diagnosis. Further, over 40% of patients with AS had moderate to severe disease, among whom the 4-year all-cause mortality ranged from 33.5% to 44.9% (14). There is an urgent need for earlier detection among patients with AS, in addition to identifying ways to improve follow up and expedite intervention (14, 15).

Identifying and monitoring patients with mitral regurgitation earlier in the disease course is similarly important. Appropriate timing for valve repair/replacement leads to better outcomes (3). This remains a challenge across the United States, but rural populations are disproportionately affected, usually because of challenges accessing surgical centers (16).

Compared to our work presented here, prior studies exploring non-invasive methods to detect VHD relied heavily on machine learning algorithms and/or sophisticated hardware.

Chorba et al. assessed the performance of a deep learning model to detect murmurs using recordings from digital stethoscopes. Compared to echocardiograms, the algorithm had a sensitivity of 93.2% and specificity of 86.0% for AS and a sensitivity of 66.2% and a specificity 94.6% for MR. Yet, the authors excluded mild AS cases from modeling and had to exclude an additional 13% of recordings due to poor quality. Had held-out recordings been included, the algorithm's sensitivity for moderate-severe AS and MR murmur detection could have been as low as 84% and 57%, respectively (17). Prince et al. similarly developed deep learning algorithms to both detect and classify different forms of SHD using digital stethoscopes (18). For all audible SHD, the algorithm performed well (sensitivity 87.7% and specificity 82.0%), and for aortic stenosis specifically, the reported sensitivity and specificity was 100% and 73.5%, respectively.

A notable limitation of this work using digital stethoscopes is limiting analyses to *audible* murmurs. Of the 409 persons with echo-confirmed SHD in the study by Prince et al., one-third had inaudible disease and were unaccounted for in their algorithm performance (18). Additionally, both Prince and Chorba limited their analyses to moderate to severe AS, excluding mild AS from model performance. Models that are restricted to moderate-to-severe, audible heart disease will inevitably fall short of the population's screening needs. The methods used in this pilot were agnostic to audible nature of the enrolled subject's condition. At no point did we “listen” to the patient or even check if a murmur had been previously documented.

More recently, Zhou et al. performed a prospective study using AI stethoscopes to develop machine learning algorithms for the detection of left VHD by processing raw sounds, and extracting features across different domains including the nonlinear domain (19). Performance was best for the collective detection of left-sided VHD (sensitivity 70.0%, AUC 75.5%) with decreased performance for aortic stenosis specific models (sensitivity 66.7%, AUC 63.3%). The authors used raw sound, therefore did not limit analyses to audible murmurs and included subjects with mild disease, the combination of which could explain the relatively poor model performance compared to previously published work. None of the Test Set models had performance characteristics that exceeded 80%. Yet, the models were overly reliant on linear features extracted from the frequency and cepstrum domains. This work and previous work by study collaborators suggests that auscultation data is chaotic and nonlinear features are better predictors of human conditions. The main limitation of the acoustic features is that they generally assume stable periodicity and linear phasal relationships that do not typically exist in chaotic systems which places unnecessary burden on model fitting.

Dhingra et al. used artificial intelligence to develop an ensemble deep learning algorithm using electrocardiographic (ECG) images to screen for structural heart disease. The AUC for the detection of aortic stenosis was 0.804 (95% CI 0.784–0.824) and 0.792 (95% CI 0.776–0.807) for mitral regurgitation (20). Similarly, Poterucha et al. developed a deep learning model (EchoNext) for the detection of SHD using 12-lead ECG's and reported an algorithm performance with strong discriminatory capacity for aortic stenosis (AUC 0.864) (21). These SHD deep learning models using ECGs performed well overall and obviate the concerns for audibility. But like many digital stethoscope models, Dhingra and Poterucha excluded mild AS and optimized the model for positive predictive value at the expense of sensitivity, limiting their future utility as a screening tool.

An additional challenge in scaling VHD detection with AI algorithms using either digital stethoscopes or ECGs is a requirement for additional, potentially sophisticated and expensive, hardware. The NLD models presented here have impressive accuracy and less data burden and can be paired with any technological hardware that can record sound. This study used unmodified mobile phones for their high recording speeds, wide detection of frequencies (including infrasonic), excellent sensitivity, and most importantly, ubiquity, with estimates approaching 8 billion mobile phones in the world (22). Sound and data collection via mobile phone significantly improves the accessibility and acceptability of the proposed application. There is a wide range of potential applications, from in-home self-screening and diagnosis to in-office front line screening and referrals. Leveraging mobile phones could democratize access to cardiovascular screening. Additionally, our models for AS and MR included mild disease and retained high performance characteristics compared to other published studies.

Nonetheless, the deep learning algorithms above show great promise and are indeed a significant improvement on current standards of care. Other studies combine spectrogram analysis of murmurs with ECG interpretation to improve AI algorithm performance for the detection of SHD (23). NLD software could further advance this important work.

In this study, we simply sought to prove that NLD variables could be mapped to echocardiogram data for specific conditions. While the models developed as part of this work could be used for the detection of structural heart diseases like AS and MR, larger studies are necessary to confirm these findings.

## Limitations

This study was limited to a single site with a relatively homogenous population. The demographic characteristics of our target populations (AS and MR) are similar to what is described elsewhere (24). Notwithstanding, external validity in different populations and contexts needs to be established. Further, the sample size was small as this first step was to establish feasibility. The Train-Test models should be viewed as a control against over-fitting and not conclusive evidence of reproducibility. Yet, we contend that the small number of subjects with outcome of interest understates the feasibility of the technology and future performance metrics could be greater. We did not meet target enrollment to develop a model for mitral regurgitation and while our *post-hoc* adapted MR model that included mild disease had promising performance, additional studies will be needed to establish feasibility. Inter-operator measurements in echocardiography and reading are an additional limitation but mitigated by our study protocol's addition of a second sonographer and cardiologist overread.

## Conclusions

Our study establishes the feasibility of using auscultation recordings collected from unmodified mobile phones and analyzed using nonlinear dynamics to identify features subsequently mapped to predict gold standard echocardiogram findings among subjects with aortic stenosis. NLD models have the potential to improve the screening, diagnosis, and monitoring of VHD and overcome existing technical, cost, and logistical barriers that remain for existing standards of care and emerging deep learning algorithms.

## Data Availability

The raw data supporting the conclusions of this article will be made available by the authors, without undue reservation.
