# Supplementary material for: Feasibility of detecting aortic stenosis with mobile phone auscultation data: a pilot study
Source: Front Cardiovasc Med. 2026 Mar 4;13:1768473. doi: 10.3389/fcvm.2026.1768473 (PMC12996200; doi:10.3389/fcvm.2026.1768473)

Supplementary Data

Supplementary table 1. Demographic elements extracted on all study subjects.

| Study ID | Name | Medical record number (MRN) | Date of birth |
| --- | --- | --- | --- |
| Biological sex (m/f) | Age (years) | Ethnicity | Race |
| Height (cm) | Weight (kg) | Body Mass Index | Body surface area (m^2^) |
| Last three Blood pressures | Last three recorded heart rates | Last three pulse oximetry values |  |
| Enrollment location (echo lab or cardiology clinic) | Cardiopulmonary admission within the past year (y/n) and date | Relevant co-morbidities and date of diagnosis:  Cardiovascular: coronary artery disease, hypertension, heart failure, valvular disease, cardiomyopathies, peripheral vascular disease including DVT, atrial arrythmias;  Pulmonary: COPD, obstructive sleep apnea, reactive airway disease, asthma (and severity), chronic lung disease, interstitial lung disease, history of pulmonary emboli | |
| Select cardiopulmonary symptoms: dyspnea at rest, exertional dyspnea, chest pain, chest tightness, cough, wheeze | Date of study diagnosis (AS, MR, Non-AS/MR SHD) |  |  |

Supplementary table 2. Echocardiogram elements extracted on all study subjects.

| Date of study | Systolic blood pressure | Diastolic blood pressure | Source (Department) of echo order (primary care, cardiology, other) |
| --- | --- | --- | --- |
| Related to left ventricular function | | | |
| Ejection fraction (ideally by Simpsons Biplane method or summarized in report) | Left ventricular inner diameter systole -- LVIDS (cm) | MV E/E1 lateral | Wall motion abnormality ? (Y/N) |
| Diastolic dysfunction (Y/N, grade I-IV) | Intra-ventricular septum (IVS) thickness (cm) | E/A ratio | Reported Ejection Fraction (%) as summarized by reading cardiologist |
| Left ventricular inner diameter diastole -- LVIDD (cm) | MV E1 tissue velocity lateral (cm/s) | Velocity time-integral, VTI (cm) |  |
| Related to right ventricular function | | | |
| RV-diastolic basal diameter (cm) | TAPSE (cm) | TR peak velocity (m/s) |  |
| Valvular function | | | |
| Tricuspid regurgitation (yes/no, mild, moderate, severe, torrential) | Pulmonic regurgitation (yes/no, mild, moderate, severe) |  |  |
| Mitral regurgitation (y/n, mild, moderate, severe) | Etiology (primary vs. secondary) | Mitral annular calcification (y/n) | Flail leaflet? (y/n) |
| Effective regurgitant orifice area (cm2) | Regurgitant fraction (%) | Regurgitant volume (mL) | Pulmonic vein systolic flow reversal (y/n) |
| Vena contracta width (cm) |  |  |  |
| Aortic regurgitation (y/n, mild, moderate, severe | AV calcification (y/n) | Aortic valve peak velocity (m/s) | AV VTI ratio |
| Aortic stenosis (y/n, mild, moderate, severe) | AV mean gradient (mmHg) | AV area by continuity equation VTI (cm2) |  |
| Bicuspid/tricuspid aortic valve | AV peak gradient (mmHg) | SVI (stroke volume index) |  |
| Other | | | |
| Left atrial volume index (ml/m2) |  |  |  |

Supplementary Table 3 – Cardiopulmonary co-morbidities among all subjects and stratified by enrollment group.

| Variable | All | Aortic stenosis | Mitral Regurgitation | Structural heart disease, Other | Controls |
| --- | --- | --- | --- | --- | --- |
| Subjects – N (%) | 248 (100) | 69 (27.8) | 24 (9.7) | 73 (29.4) | 82 (33.1) |
| None | 14 (5.7) | 1 (1.5) | 3 (12.5) | 2 (2.7) | 8 (9.8) |
| Coronary Artery Disease | 108 (43.6) | 35 (50.7) | 12 (50.0) | 36 (49.3) | 25 (30.5) |
| Hypertension | 182 (73.4) | 56 (81.2) | 19 (79.2) | 52 (71.2) | 55 (67.1) |
| Systolic Heart Failure | 39 (15.7) | 4 (5.8) | 2 (8.3) | 27 (37.0) | 6 (7.3) |
| Diastolic Heart Failure | 48 (19.4) | 11 (15.9) | 5 (20.8) | 20 (27.4) | 12 (14.6) |
| Valvular disease (Not AS/MR)^A^ | 53 (21.4) | 16 (23.2) | 8 (33.3) | 22 (30.1) | 7 (8.5) |
| Cardiomyopathy | 46 (18.6) | 5 (7.3) | 5 (20.8) | 28 (38.4) | 8 (9.8) |
| Peripheral Vascular Disease | 24 (9.7) | 11 (15.9) | 1 (4.2) | 7 (9.6) | 5 (6.1) |
| Venous Thromboembolism | 9 (3.6) | 3 (4.4) | 0 (0) | 4 (5.5) | 2 (2.4) |
| Atrial Arrythmias | 77 (31.1) | 16 (23.2) | 8 (33.3) | 31 (42.5) | 22 (26.8) |
| Ventricular Arrythmias | 12 (4.8) | 1 (1.5) | 1 (4.2) | 5 (6.9) | 5 (6.1) |
| Aortic Dilation | 65 (26.2) | 17 (24.6) | 6 (25.0) | 21 (28.8) | 21 (25.6) |
| Other cardiovascular* | 20 (8.1) | 8 (11.6) | 1 (4.2) | 7 (9.6) | 4 (8.9) |
| Chronic obstructive pulmonary disease | 20 (8.1) | 3 (4.4) | 3 (12.5) | 6 (8.2) | 8 (9.8) |
| Obstructive sleep apnea | 55 (22.2) | 12 (17.4) | 10 (41.7) | 17 (23.3) | 16 (19.5) |
| Reactive airway disease or asthma | 30 (12.1) | 8 (11.6) | 4 (16.7) | 7 (9.6) | 11 (13.4) |
| Interstitial lung disease | 6 (2.4) | 4 (5.8) | 0 (0.0) | 2 (2.7) | 6 (2.4) |
| Chronic lung disease, other | 7 (2.8) | 1 (1.5) | 1 (4.2) | 2 (2.7) | 3 (3.7) |
| Pulmonary embolic history | 8 (3.2) | 2 (2.9) | 0 (0.0) | 2 (2.7) | 4 (4.9) |
| Pulmonary hypertension | 12 (4.8) | 3 (4.4) | 3 (12.5) | 2 (2.7) | 4 (4.9) |
| Other pulmonary history | 3 (1.2) | 1 (1.5) | 0 (0.0) | 1 (1.4) | 1 (1.2) |
| ^A^ Mild or trivial mitral regurgitation (MR) was documented among 21, 29, 2 subjects with AS, Other SHD, and controls, respectively but not included in the “other valvular disease” category to avoid confusion with mitral regurgitation group.  * Other conditions included AV or bundle branch block (9), pulmonary hypertension (3), pericardial effusion (3), cardiac sarcoidosis (2), atrial septal defect (2), congenital coronary syndrome (1), and dissection (1).  ** Other pulmonary conditions included pulmonary sarcoidosis (2), and diaphragmatic paralysis (1) | | | | | |

Supplementary Figure 1. Maximal Lyapunov exponent and correlation dimension of study staff (n=248) and subject (n=248) aortic site auscultation recordings reveal presence of chaos and low dimensionality.


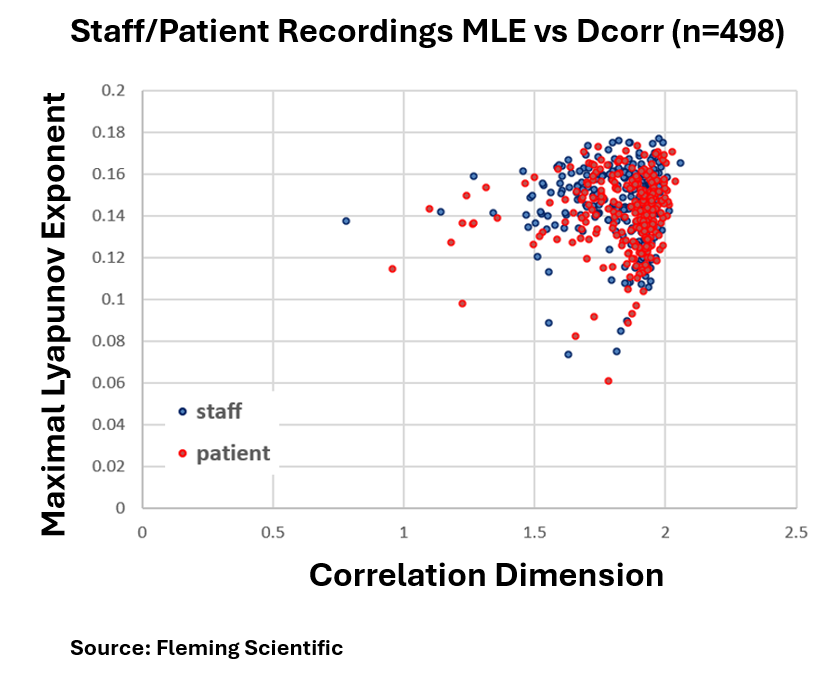

Supplement: Supplementary file 1 [file Datasheet1.docx]
